# Subgroups of adult-onset diabetes: a data-driven cluster analysis in a Ghanaian population

**DOI:** 10.1038/s41598-023-37494-2

**Published:** 2023-07-04

**Authors:** Ina Danquah, Isabel Mank, Christiane S. Hampe, Karlijn A. C. Meeks, Charles Agyemang, Ellis Owusu-Dabo, Liam Smeeth, Kerstin Klipstein-Grobusch, Silver Bahendeka, Joachim Spranger, Frank P. Mockenhaupt, Matthias B. Schulze, Olov Rolandsson

**Affiliations:** 1 Heidelberg Institute of Global Health (HIGH), Faculty of Medicine and University Hospital, Heidelberg University, Heidelberg, Germany; 2 German Institute for Development Evaluation (DEval), Bonn, Germany; 3 Department of Medicine, University of Washington, Seattle, WA USA; 4 Center for Research on Genomics and Global Health, National Human Genome Research Institute, National Institutes of Health, Bethesda, MD USA; 5 Department of Public Health, Amsterdam UMC, location AMC, University of Amsterdam, Amsterdam, The Netherlands; 6 Kwame Nkrumah University of Science and Technology (KNUST), Kumasi, Ghana; 7 London School of Hygiene and Tropical Medicine (LSHTM), London, UK; 8 Julius Global Health, Julius Center for Health Sciences and Primary Care, University Medical Center Utrecht, Utrecht University, Utrecht, The Netherlands; 9 Division of Epidemiology and Biostatistics, School of Public Health, Faculty of Health Sciences, University of the Witwatersrand, Johannesburg, South Africa; 10 MKPGMS-Uganda Martyrs University, Kampala, Uganda; 11 Department of Endocrinology and Metabolism, Charité - Universitaetsmedizin Berlin, Corporate Member of Freie Universitaet Berlin, Humboldt-Universitaet zu Berlin, Berlin Institute of Health, Berlin, Germany; 12 Institute of Tropical Medicine and International Health, Charité - Universitaetsmedizin Berlin, Corporate Member of Freie Universitaet Berlin, Humboldt-Universitaet zu Berlin, and Berlin Institute of Health, Berlin, Germany; 13 Department of Molecular Epidemiology, German Institute of Human Nutrition Potsdam-Rehbruecke, Nuthetal, Germany; 14 Institute of Nutritional Science, University of Potsdam, Nuthetal, Germany; 15 Department of Public Health and Clinical Medicine, Section of Family Medicine, Umeå University, Umeå, Sweden

**Keywords:** Epidemiology, Type 2 diabetes

## Abstract

Adult-onset diabetes mellitus (here: aDM) is not a uniform disease entity. In European populations, five diabetes subgroups have been identified by cluster analysis using simple clinical variables; these may elucidate diabetes aetiology and disease prognosis. We aimed at reproducing these subgroups among Ghanaians with aDM, and establishing their importance for diabetic complications in different health system contexts. We used data of 541 Ghanaians with aDM (age: 25–70 years; male sex: 44%) from the multi-center, cross-sectional Research on Obesity and Diabetes among African Migrants (RODAM) Study. Adult-onset DM was defined as fasting plasma glucose (FPG) ≥ 7.0 mmol/L, documented use of glucose-lowering medication or self-reported diabetes, and age of onset ≥ 18 years. We derived subgroups by cluster analysis using (i) a previously published set of variables: age at diabetes onset, HbA1c, body mass index, HOMA-beta, HOMA-IR, positivity of glutamic acid decarboxylase autoantibodies (GAD65Ab), and (ii) Ghana-specific variables: age at onset, waist circumference, FPG, and fasting insulin. For each subgroup, we calculated the clinical, treatment-related and morphometric characteristics, and the proportions of objectively measured and self-reported diabetic complications. We reproduced the five subgroups: cluster 1 (obesity-related, 73%) and cluster 5 (insulin-resistant, 5%) with no dominant diabetic complication patterns; cluster 2 (age-related, 10%) characterized by the highest proportions of coronary artery disease (CAD, 18%) and stroke (13%); cluster 3 (autoimmune-related, 5%) showing the highest proportions of kidney dysfunction (40%) and peripheral artery disease (PAD, 14%); and cluster 4 (insulin-deficient, 7%) characterized by the highest proportion of retinopathy (14%). The second approach yielded four subgroups: obesity- and age-related (68%) characterized by the highest proportion of CAD (9%); body fat-related and insulin-resistant (18%) showing the highest proportions of PAD (6%) and stroke (5%); malnutrition-related (8%) exhibiting the lowest mean waist circumference and the highest proportion of retinopathy (20%); and ketosis-prone (6%) with the highest proportion of kidney dysfunction (30%) and urinary ketones (6%). With the same set of clinical variables, the previously published aDM subgroups can largely be reproduced by cluster analysis in this Ghanaian population. This method may generate in-depth understanding of the aetiology and prognosis of aDM, particularly when choosing variables that are clinically relevant for the target population.

## Introduction

The worldwide upsurge of diabetes mellitus is mainly attributed to increases of adult-onset diabetes mellitus (aDM) in low- and middle-income countries^1^. This comprises mainly type 2 diabetes, Latent Autoimmune Diabetes in Adults (LADA), and type 1 diabetes, and translates to 75% of all diabetes cases worldwide^2^. Among adults in Ghana and Ghanaian migrants in Europe, aDM is highly prevalent, ranging between 4 and 15%^3^. Importantly, there are ethnic differences in the risk of diabetic complications. A recent systematic literature review demonstrates that individuals with diabetes mellitus of African ancestry are at significantly higher risk of diabetic complications than European populations, such as end-stage renal disease^4^. At the same time, geographic context plays an important role for disease management and the prevalence of diabetic complications. Diabetes awareness, appropriate treatment, and good glycemic control are lower in non-migrants compared to Ghanaian migrants^5^. Non-migrant Ghanaians show higher prevalence of diabetic complications than Ghanaian migrants in Europe^6^. As a consequence, productivity and quality of life decrease among non-migrant Ghanaians diagnosed with aDM, translating into increased healthcare expenditure and elevated risk for premature mortality^7^.

The classification into aDM subgroups based on clinical and anthropometric characteristics is a promising approach to understanding the disease aetiology and possibly progression^8,9^. Ahlqvist et al.^9^ have performed a hierarchical cluster analysis with six common clinical variables and have identified five subgroups among Scandinavian adults with newly diagnosed diabetes mellitus. These subgroups are characterized by distinct clinical and anthropometric features and show marked differences in the development of diabetic complications. Since then, several research groups have replicated this approach. In 2020, Sarria-Santamera et al.^10^ have synthesized the findings of 14 studies that identified subgroups by unsupervised learning methods. Five of them have applied hierarchical clustering^8,11–13^, and five subgroups are common^10^: severe autoimmune diabetes (SAID), severe insulin-deficient diabetes (SIDD), severe insulin-resistant diabetes (SIRD), mild obesity-related diabetes (MORD), and mild age-related diabetes (MARD). Regarding complications, SIRD shows increased risk for kidney disease in some^9,11–13^, but not all studies^14,15^. SIDD and partly SAID show increased risks for retinopathy. The results are conflicting for proportions of macrovascular complications^10^. In summary, these studies suggest that cluster analysis can be useful for identifying aDM subgroups early in the course of the disease, and thereby, contributing to understanding disease aetiology. Still, the verification of this approach is pending in more diverse populations and with variables that have established clinical relevance for them. For instance, anthropometric measures reflecting fat distribution might be more suitable than BMI in sub-Saharan African populations^16^. So far, studies in African-descent populations are scarce^17^, and virtually absent for populations from sub-Saharan Africa.

Therefore, the aim of this study was to establish the reproducibility of aDM subgroups by means of cluster analysis in a Ghanaian study population. The specific objectives were (i) to apply the previously published method by Ahlqvist et al. for the identification of aDM subgroups^9^, (ii) to generate subgroups using Ghana-specific clinical variables, and (iii) to determine the proportions of diabetic complications among the identified subgroups.

## Methods

### Study design and population

The Research on Obesity and Diabetes among African Migrants (RODAM) study is a multi-center cross-sectional study that was conducted between July 2012 and September 2015. It involved Ghanaian adults (25–70 years) living in rural and urban Ghana (Ashanti region), and three European cities (Amsterdam, London, and Berlin) (N = 6385)^3^, with a crude prevalence of aDM of 9%^18^. The primary objective of the RODAM study was to disentangle the relative contributions of (epi)genetic and non-genetic risk factors for obesity and diabetes mellitus. The study protocol and the procedures of the RODAM study have previously been published^19^. Medical history, lifestyle and socio-economic factors were recorded either by ethnically matched personnel in questionnaire-based interviews or were self-administered. Specifically, we included questions about the age of disease onset, previous diagnosis of diabetes mellitus, the start of medication prescription, and the type of glucose-lowering medications.

### Biomaterial collection and laboratory measurements

Adult-onset diabetes mellitus was defined according to WHO guidelines as fasting plasma glucose ≥ 7.0 mmol/L, documented use of glucose-lowering medication or self-reported diabetes. We excluded individuals with age of disease onset before 18 years. Fasting venous blood samples and urine samples were collected by trained personnel according to standard operating procedures; blood samples were centrifuged. Urine, serum and plasma samples were ultimately stored at − 80 °C. All biochemical analyses were performed using an ABX Pentra 400 chemistry analyser (ABX Pentra; Horiba ABX, Germany).

The biomarker profile of this study comprised HbA1c, serum insulin, blood lipids (total cholesterol, triglyceride, high-density lipoprotein cholesterol, low-density lipoprotein cholesterol), inflammatory markers (C-reactive protein), and liver enzymes (aspartate aminotransferase (AST), alanine aminotransferase (ALT), and γ-glutamyl transferase (GGT)). We calculated the Homeostatic Model Assessment for insulin resistance (HOMA-IR) and for beta-cell capacity (HOMA-beta) according to Matthews et al.^20^; using fasting plasma glucose and fasting insulin in the assessments. Serum creatinine concentration was determined by a kinetic colorimetric spectrophotometric isotope dilution mass spectrometry calibration method (Roche Diagnostics). Estimated glomerular filtration rate (eGFR) was calculated using the 2009 CKD-EPI (CKD Epidemiology Collaboration) creatinine equation and the severity of kidney disease categorized according to the 2012 KDIGO guidelines^21^. Urinary albumin concentration (in mg/L) was measured by an immunochemical turbidimetric method (Roche Diagnostics). Urinary creatinine concentration (in μmol/L) was measured by a kinetic spectrophotometric method (Roche Diagnostics). Urinary albumin–creatinine ratio (ACR; expressed in mg/g) was calculated by taking the ratio between urinary albumin and urinary creatinine and stratified according to the 2012 KDIGO classifications: A1, < 3 mg/mmol (normal to mildly increased); A2, 3 to 30 mg/mmol (moderately increased); and A3, > 30 mg/mmol (severely increased).

Antibodies against glutamic acid decarboxylase (GAD65Ab) were determined by radioligand binding assay (RBA) using established cut-offs (Ghana: > 121 U/mL; Europe: > 97 U/mL), as previously described by Hampe et al.^22^.

### Physical examinations

Trained study personnel performed the physical examinations. Anthropometric measurements were taken in light clothes and without shoes, including weight (kg) by a person scale, height (cm) by a stadiometer, and waist circumference (cm) using a measuring tape (all devices SECA, Germany). We calculated Body Mass Index (BMI) as weight over squared height (kg/m^2^). Blood pressure (BP) was measured three times using a validated semiautomated device (MicrolifeWatch BP home, Widnau, Switzerland), with appropriately sized cuffs after at least 5 min rest while seated. The mean of the last two BP measurements was used for the analyses. Hypertension was defined as systolic BP ≥ 140 mmHg and/or diastolic BP ≥ 90 mmHg, and/or being on antihypertensive medication treatment. Ankle brachial index (ABI), the ratio of the resting systolic blood pressure at the ankle to the resting systolic brachial pressure at the arm, was obtained from two blood pressure measurements on the left side (leg and arm) and two on the right side (leg and arm) using the Microlife Watch BP Office ABI with appropriately sized cuffs, after at least 10 min of supine rest. The cuffs for measuring the ankle and brachial pressures were placed just above the ankle and at the upper arm, respectively.

### Definitions of diabetic complications

Validated methods were used to detect kidney dysfunction, peripheral artery disease (PAD) and coronary artery disease (CAD). Albuminuria was defined as urinary albumin ≥ 3 mg/mmol, and impaired eGFR was based on CKD-EPI eGFR criteria, comprising G3 to G5. Nephropathy was defined as albuminuria or microalbuminuria (stages 2–4) according to the report from Joint Committee on Diabetic Nephropathy^23^. PAD was defined as ankle brachial index (ABI) < 0**.**9^24^. CAD was assessed using the WHO Rose angina questionnaire^25^. We used self-reported data for retinopathy and stroke. Retinopathy was assessed by a positive response to the question ‘Have you ever been told by a doctor that you have eye disease or eye damage as a result of diabetes?’. Stroke was assessed by a positive reply to the question ‘Have you ever had a stroke?’.

### Statistical analysis

All analyses were conducted using the software SAS, version 9.3 (SAS Institute, Cary, North-Carolina, USA).

### Missing data handling

Among the 541 participants with aDM, there were 140 individuals with missing or implausible values (< 2nd and ≥ 98th percentile) in any of the variables of interest. These variables comprised socio-economic data (n = 140), anthropometric measures (n = 52), biochemical data (n = 40), and lifestyle information (n = 24). To avoid selection bias and to improve statistical power, we applied multiple imputation using the discriminant fully conditional specification (FCS) method and derived 10 imputed datasets. FCS is also known as multiple imputation by chained equations (MICE) and uses separate conditional univariate imputation models specified for each incomplete variable, with other variables as predictors. Multiple imputation was applied under the assumption that the propensity of missing data can be explained by the observed data (missing at random, MAR). This assumption was supported by the good imputation efficiency (98.2–99.9%).

### Cluster analysis

Two approaches were applied to derive aDM subgroups using exploratory cluster analysis. First, we re-constructed the clusters by Ahlqvist et al.^9^ using the same six clinical and anthropometric variables (first approach). These have been chosen because they are readily available in routine clinical practice: age at onset of diabetes mellitus, body mass index (BMI), HbA1c, HOMA-beta, HOMA-IR, and the presence of glutamate decarboxylase antibodies (GAD65Ab). We centered all variables to a mean value of 0 and a standard deviation (SD) of 1. Then, a two-step cluster analysis was applied, in which the first step extracted a predefined number of clusters on the basis of silhouette width (n = 5). This step used log-likelihood as a distance measure and Schwarz’s Bayesian criterion for clustering. The second step involved k-means clustering to confirm the stability of clusters. In the k-means clustering, GAD65Ab presence was not included, because this analysis can only accommodate continuous variables. Cluster labels were assigned according to previously published subgroups by Ahlqvist et al.^9^.

Second, we repeated the cluster analysis using variables that have established clinical relevance for aDM among Ghanaian populations (second approach). More specifically, we have previously seen that anthropometric measures of abdominal obesity have the best discriminative ability for diabetes mellitus among Ghanaian adults^26,27^, and thus, waist circumference was preferred over BMI. Also, we included FPG as a readily available biomarker in resource-poor settings. Calculations of HOMA-beta and HOMA-IR based on C-peptide do not reflect the important role of serum insulin in aDM. While C-peptide only describes pancreatic insulin release, fasting insulin also reflects impaired hepatic insulin clearance, which likely contributes to insulin resistance^28^. Therefore, we preferred insulin values over C-peptide-based calculations of HOMA. In the second approach, we did not use GAD65Ab, because our previous work in the RODAM Study suggests that GAD65Ab may poorly discriminate for diabetes mellitus^22^ and may not be readily available in sub-Saharan Africa. Age at onset of aDM was also included in this second approach. The same two-step cluster analysis was applied to the data as described above, with the modification that 2 to 5 cluster solutions were generated in step 1. The optimal number of clusters to be extracted was identified based on cluster size (> 5% of the population) and the dendrogram of explained variance (R^2^ > 0**.**25). Cluster labels were assigned by examining the phenotypic characteristics.

Two sensitivity analyses were performed. First, we repeated the cluster analysis after exclusion of individuals on insulin treatment to assess the stability of the subgroups. Second, we derived clusters stratified by study location (Ghana and Europe).

### Characterization of clusters and proportions of diabetic complications

For each cluster description, we calculated means and SD for normally distributed variables, medians and interquartile ranges (IQR) for skewed variables, and proportions for categorical variables. The proportions of diabetic complications were calculated for each cluster and were compared by χ^2^-test.

### Ethics statement

The RODAM Study was conducted according to the guidelines laid down in the 1964 Declaration of Helsinki and its later amendments. All procedures involving human subjects were reviewed and approved by the respective ethics committees in Ghana (Committee on Human Research, Publication and Ethics, Kwame Nkrumah University of Science and Technology, Kumasi), the Netherlands (Medical Ethics Review Committee, Academic Medical Centre, University of Amsterdam), the UK (Observational/ Interventions Research Ethics Committee, London School of Hygiene and Tropical Medicine), and Germany (Ethics Commission, Charité—Universitaetsmedizin Berlin). Written informed consent was obtained from all participants.

### Role of the funding source

The funders of the study had no role in study design, data collection, data analysis, data interpretation, or writing of the manuscript and in the decision to submit the paper for publication.

## Results

### General characteristics of the study population

Supplementary Table S1 shows the characteristics of the total study population (N = 5898), stratified by diabetes status. In brief, participants with aDM (n = 541) had a mean age of 53.2 years (SD: 9.5 years), and 56% were women. Most individuals with aDM lived in Amsterdam (32%), followed by urban Ghana (25%), London (19%), Berlin (13%) and rural Ghana (10%). Two-thirds had none or only elementary formal education and had manual occupations; 14% were smokers. The median duration of aDM was 5 years (IQR: 1–11 years), and many participants with aDM had a family history of diabetes (41%). The mean HbA1c was 7.7% (SD: 2**.**2%). The proportion of individuals with GAD65Ab-positivity was similar between participants with diabetes mellitus and individuals without the disease (5% versus 6%). More than two-thirds of individuals with aDM were overweight or obese (BMI ≥ 25**.**0 kg/m^2^) and 56% had abdominal obesity (waist circumference > 102 cm for men and > 88 cm for women).

### Subgroups and their characteristics

**Figure 1:**
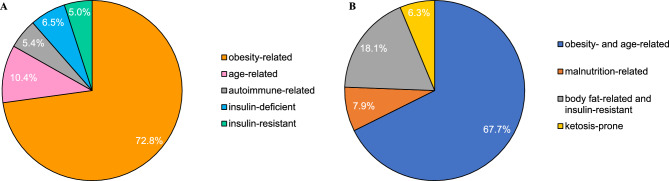
Distributions of diabetes subgroups derived by cluster analysis (**A**) using six common clinical and anthropometric variables according to Ahlqvist et al. (2018), and (**B**) using five variables with clinical relevance for Ghanaian populations.

**Figure 2:**
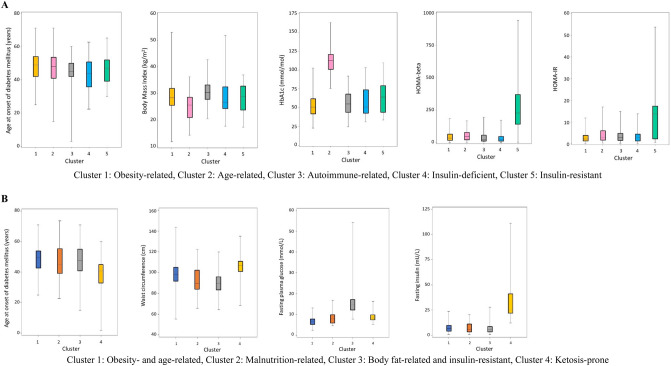
Cluster characteristics in 541 Ghanaian individuals with adult-onset diabetes (**A**) using six common clinical and anthropometric variables according to Ahlqvist et al. (2018), and (**B**) using five variables with clinical relevance for Ghanaian populations.

Figure 1 displays the proportions of aDM subgroups derived by cluster analyses using two sets of clinical and anthropometric variables. Figure 1A shows the results for the first approach, based on age of disease onset, BMI, HbA1c, HOMA-beta, HOMA-IR, and the presence of GAD65Ab; Fig. 1B depicts the identified subgroups from the second approach, based on age of disease onset, waist circumference, FPG, and insulin. The distributions of cluster characteristics are shown in Fig. 2, and the detailed characteristics of the identified subgroups are presented in Table 1.

**Table 1:** Clinical and anthropometric characteristics of adult-onset diabetes subgroups by two different cluster analysis approaches among 541 Ghanaian adults.

Characteristics	Approach 1: age at onset, BMI, HbA1c, HOMA-IR, HOMA-beta, GAD65Ab					Approach 2: age at onset, waist circumference, FPG, insulin			
	Cluster 1	Cluster 2	Cluster 3	Cluster 4	Cluster 5	Cluster 1	Cluster 2	Cluster 3	Cluster 4
	Obesity-related	Age-related	Autoimmune-related	Insulin-deficient	Insulin-resistant	Obesity- and age-related	Malnutrition-related	Body fat-related and insulin-resistant	Ketosis-prone
N	394	56	29	35	27	366	43	98	34
Age (years)	53.4 ± 9.3	53.1 ± 9.0	53.9 ± 10.2	53.7 ± 10.6	50.0 ± 11.8	53.4 ± 9.5	51.9 ± 11.7	53.4 ± 8.8	52.4 ± 8.4
Sex (male)	46.2	33.9	48.3	45.7	29.6	47.3	48.8	30.6	44.1
*Clinical*									
Age at diabetes diagnosis (years)	48.6 ± 9.1	47.7 ± 10.1	43.8 ± 11.7	42.4 ± 7.8	46.6 ± 10.0	46.0 ± 12.3	44.9 ± 13.3	46.1 ± 13.9	46.2 ± 11.3
Family history of diabetes (yes)	40.9	43.6	37.9	43.7	45.2	40.5	43.5	42.7	45.0
Diabetes medication									
Insulin	15.0	14.6	15.9	19.7	11.9	15.6	15.8	12.1	19.1
Glucose-lowering drugs*	77.5	73.8	85.9	84.6	90.0	78.6	77.7	77.9	81.8
Diet and physical activity	52.7	59.6	55.9	58.0	71.9	53.1	55.8	63.7	47.9
Insulin treatment immediately	48.5	51.8	50.0	58.0	46.7	49.3	42.8	46.2	50.3
Insulin treatment in first 6 months	27.7	32.1	30.0	41.1	36.3	27.3	30.9	31.1	33.8
Fasting glucose (mmol/L)	7.4 (5.9–9.9)	7.5 (5.5–11.8)	8.5 (6.0–11.4)	7.7 (6.0–11.6)	7.3 (4.2–8.5)	7.3 (5.7–9.6)	7.4 (6.0–10.8)	8.1 (5.7–11.1)	8.1 (5.8–12.3)
HbA1c (mmol/mol)	50.6 (42.0–62.0)	52.9 (42.4–74.6)	54.9 (44.1–68.1)	51.0 (43.1–74.0)	63.9 (44.1–79.3)	52.6 (44.0–69.4)	52.6 (40.9–73.9)	57.2 (43.8–74.0)	56.8 (45.2–84.0)
HbA1c (%)	7.1 (6.2–8.4)	7.0 (6.0–9.0)	8.1 (6.3–9.8)	7.4 (6.4–9.7)	6.7 (6.2–8.8)	7.0 (6.2–8.5)	7.0 (5.9–8.9)	7.4 (6.2–8.9)	7.3 (6.3–9.8)
Insulin (mU/L)	6.5 (4.3–9.7)	8.2 (5.5–12.3)	6.4 (4.4–10.4)	5.5 (3.5–9.4)	28.6 (11.3–44.1)	6.7 (4.4–9.7)	6.0 (3.5–9.5)	8.5 (4.7–13.4)	8.3 (4.3–11.7)
HOMA-IR	2.32 (1.29–3.87)	2.84 (1.71–6.04)	2.63 (1.54–4.86)	2.78 (1.22–4.43)	13.39 (2.25–17.26)	2.33 (1.33–3.84)	2.78 (1.15–3.95)	3.16 (1.54–6.61)	2.90 (1.22–5.38)
HOMA-beta	36.2 (18.4–63.4)	41.4 (22.5–77.9)	20.3 (12.0–55.8)	24.2 (13.4–45.6)	228.3 (142.0–370.8)	38.6 (18.7–70.4)	25.1 (17.2–68.0)	41.0 (22.4–68.8)	29.6 (11.6–78.0)
GAD65Ab (positive)	0.0	0.0	100	0.0	0.0	6.3	4.7	3.1	2.9
Urinary ketones (positive)	2.8	8.9	0.0	2.9	7.7	2.7	2.3	6.2	5.9
Hypertension (yes)	78.2	73.2	75.9	71.4	74.1	79.0	76.7	70.4	73.5
ASAT (U/L)	29.7 (24.2–36.4)	33.2 (26.2–41.0)	31.3 (23.4–38.7)	29.0 (23.5–38.8)	30.6 (25.2–27.6)	28.9 (24.1–36.3)	29.0 (23.5–41.2)	33.2 (26.9–40.7)	30.3 (25.6–38.9)
ALAT (U/L)	21.0 (16.3–27.6)	25.0 (15.6–32.3)	20.9 (16.3–29.4)	20.8 (15.8–25.8)	21.9 (15.5–32.3)	21.1 (16.3–27.6)	20.4 (14.6–25.8)	22.2 (16.1–29.7)	20.5 (16.9–31.7)
ASAT/ALAT	1.39 (1.16–1.73)	1.43 (1.09–1.74)	1.45 (1.17–1.73)	1.55 (1.25–1.90)	1.34 (1.03–1.65)	1.37 (1.14–1.72)	1.56 (1.31–1.85)	1.44 (1.20–1.83)	1.32 (1.19–1.56)
GGT (U/L)	37.9 (27.7–53.7)	40.8 (32.2–57.9)	40.7 (29.1–62.2)	36.4 (29.0–47.6)	37.1 (26.4–51.3)	38.9 (28.7–53.7)	36.4 (26.6–44.1)	37.7 (27.9–55.7)	41.2 (27.2–58.3)
*Anthropometric*									
Body mass index (kg/m^2^)	28.9 ± 5.3	25.2 ± 5.1	31.0 ± 5.0	29.0 ± 7.2	28.5 ± 5.4	28.7 ± 5.5	26.5 ± 6.3	29.3 ± 4.9	28.7 ± 6.1
Waist circumference (cm)	97.0 ± 12.8	97.6 ± 13.9	96.6 ± 11.7	93.8 ± 14.2	102.3 ± 11.7	97.3 ± 13.1	93.3 ± 14.6	97.9 ± 12.0	97.3 ± 11.0
Fat-free mass (kg)	52.5 ± 9.7	50.8 ± 7.9	52.3 ± 10.3	51.2 ± 10.7	54.1 ± 7.9	52.8 ± 9.7	51.5 ± 11.3	50.6 ± 8.5	52.8 ± 7.6
Fat mass (kg)	25.1 ± 10.6	27.7 ± 11.3	23.2 ± 10.8	22.4 ± 11.4	30.6 ± 12.2	25.1 ± 10.9	21.7 ± 10.9	27.6 ± 10.2	25.7 ± 12.1
Fat percentage (%)	31.7 ± 8.6	34.3 ± 9.3	29.9 ± 9.7	29.7 ± 8.4	35.2 ± 8.0	31.5 ± 8.7	29.0 ± 8.2	34.5 ± 8.4	31.9 ± 9.6

Data are presented as means ± standard deviations, medians (interquartile ranges) or percentages. ALAT, alanin-aminotransferase; ASAT, aspartate-aminotransferase; BMI, body mass index; FPG, fasting plasma glucose; GAD65Ab, glutamic-acid decarboxylase dehydrogenase 65 auto-antibody; GGT, gamma-glutamyl-transferase; HOMA-IR, homeostasis model assessment of insulin resistance; HOMA-beta, homeostasis model assessment of beta-cell function. * comprises all medication with ATC codes A10.

In the first approach, we reproduced the five subgroups reported by Ahlqvist et al.^9^: cluster 1 (obesity-related, 73%), cluster 2 (age-related, 10%), cluster 3 (autoimmune-related, 5%), cluster 4 (insulin-deficient, 7%), and cluster 5 (insulin-resistant, 5%). The obesity-related subgroup was characterized by late age of disease onset. The age-related subgroup also showed late age of disease onset and high frequency of female cases. The autoimmune-related subgroup (GAD65Ab-positive) had a younger age at disease onset and the least frequent family history of diabetes. The insulin-deficient subgroup was characterized by early age of disease onset, and the lowest serum insulin levels. For the insulin-resistant subgroup, the majority of cases were women with the highest serum insulin levels, HOMA-IR and HOMA-beta. The stability of subgroups according to k-means clustering was robust, except for the discrimination between autoimmune and insulin-deficient subgroups. These were extracted as one subgroup by k-means analysis (Table S2).

In the second approach, we identified four subgroups: cluster 1 (obesity- and age-related, 68%), cluster 2 (malnutrition-related, 8%), cluster 3 (body fat-related and insulin-resistant, 18%), and cluster 4 (ketosis-prone, 6%). Despite the fact that we omitted GAD65Ab from this cluster analysis, 23 out of the 29 individuals with GAD65Ab clustered in the ketosis-prone subgroup. The malnutrition-related subgroup had the lowest serum insulin concentration and HOMA-beta, and showed the lowest BMI, waist circumference and fat mass. The body fat-related and insulin-resistant subgroup was characterized by female preponderance, high fasting glucose concentration, and high serum insulin levels. This was also seen for the ketosis-prone subgroup, who also showed high BMI and waist circumference; 6% of them had urinary ketone bodies. Again, the stability of subgroups was confirmed through k-means clustering, with some overlap between the obesity- and age-related subgroup and the body fat-related and insulin-resistant subgroup (Table S2).

Overlaps between the two approaches are presented in Supplementary Figure S1. Most individuals in the obesity-related subgroup of the first approach remained in the obesity- and age-related subgroup of the second approach. Individuals who were clustered in the age-related subgroup (first approach) transitioned to the body fat-related and insulin-resistant subgroup (second approach). Also, most participants in the insulin-deficient subgroup (first approach) were relocated to the malnutrition-related subgroup (second approach).

### Medication and diabetic complications by subgroups

**Figure 3:**
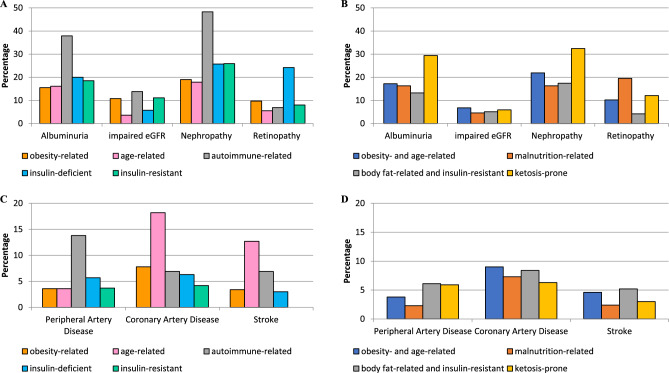
Microvascular (upper panel) and macrovascular complications (lower panel) among diabetes subgroups derived from six common clinical and anthropometric variables (**A**,**C**) and from five variables with clinical relevance for African descent populations (**B**,**D**). None of the percentages were significantly different from those in the first cluster, according to χ2-test.

We describe the distributions of medications across subgroups in Table 1 and present the proportions of diabetic complications by subgroup in Fig. 3. The majority of study participants received—with overlap—oral glucose-lowering medication (79%), followed by lifestyle modifications (55%), and insulin injections (15%). Almost half of the participants with aDM received insulin treatment immediately after diagnosis (48%), and one-third during the first 6 months after diagnosis (Table 1). Regarding diabetic complications, microvascular conditions (retinopathy, nephropathy, albuminuria) were more common than macrovascular complications (Coronary Artery Disease (CAD), Peripheral Artery Disease (PAD), stroke; Fig. 3). Kidney disease was most prominent with about one-third of the participants who fulfilled the criteria for nephropathy. This was followed by self-reported retinopathy amounting to 14% in the total study population. Macrovascular complications were seen in 7% for estimated CAD, 4% for PAD, and 3% for self-reported stroke.

Among the subgroups derived from the first approach, the following diabetic complications were observed (Fig. 3A,C). The age-related subgroup showed the highest proportions of estimated CAD (18%) and self-reported stroke (13%). Liver enzymes were increased in this subgroup (Table 1). In the autoimmune-related subgroup, markers of kidney dysfunction and PAD were more prominent than in other subgroups. The insulin-deficient subgroup showed the highest proportions of retinopathy (24%). Yet, neither the proportions of complications nor the distributions of diabetes medication were significantly different between the subgroups.

The four subgroups derived from the second approach were characterized as follows (Fig. 3B,D). The ketosis-prone subgroup showed preponderance of albuminuria and nephropathy. The malnutrition-related subgroup had the highest proportion of retinopathy. Yet again, none of the between-group differences were statistically significant. Regarding medications, almost two-thirds of the body fat-related and insulin-resistant subgroup received diet and physical activity, while this applied to less than half of the ketosis-prone subgroup. Both, insulin and glucose-lowering medication, were most frequently prescribed in the ketosis-prone subgroup (Table 1).

### Sensitivity analyses

We performed two sensitivity analyses. First, we repeated the second cluster analysis approach to account for the influence of insulin treatment on serum insulin concentration. For this analysis, 87 out of 541 individuals who were on insulin medication were excluded. Supplementary Table S3 shows the corresponding subgroups. Again, the largest cluster was the obesity- and age-related subgroup (40%), followed by malnutrition-related diabetes (39%), body fat-related and insulin-resistant diabetes (18%), and ketosis-prone diabetes (3%).

Also, we derived aDM subgroups using the Ghana-specific set of variables and stratified by location (Ghana versus Europe) to account for contextual differences. The proportions of subgroups in the two locations are shown in Supplementary Figure S2, and their characteristics are presented in Supplementary Table S4. The subgroups derived per location were similar in their characteristics but differed in proportions. Malnutrition-related diabetes was more common in Ghana (14%) than in Europe (1%), while this was the opposite for the obesity- and age-related subgroup (Ghana: 29%; Europe: 47%). The proportions of ketosis-prone diabetes and the body fat-related and insulin-resistant subgroup were similar between Ghana and Europe (Table S4). Finally, the distributions of complications across the site-specific subgroups are presented in Supplementary Figure S3. In Ghana and Europe, kidney dysfunction was most prevalent in the subgroups characterized by insulin-resistance. Macrovascular complications were rather common in ketosis-prone diabetes. The occurrence of retinopathy did not dominate in any subgroup.

## Discussion

### Summary of main results

Here, we reproduced, for the first time among sub-Saharan African adults, subgroups of aDM using data-driven cluster analysis with six simple clinical variables. We extracted the same five clusters as in the original study^9^: obesity-related (73%), age-related (10%), autoimmune-related (5%), insulin-deficient (7%), and insulin-resistant (5%). In comparison to European and Asian populations^10^, there were marginal differences in the characteristics of subgroups, their proportions, and the distributions of diabetic complications across clusters. In a second approach, we derived subgroups by employing variables with established clinical relevance for Ghanaian populations. This approach yielded four subgroups that had only some overlap with the five initial clusters regarding clinical, treatment-related and complication profiles: obesity- and age-related (68%), malnutrition-related (18%), body fat-related and insulin-resistant (8%), and ketosis-prone (6%).

### Reproducibility of subgroups

The first approach generated similar clusters compared to those initially discovered in Scandinavian populations^9^. However, the RODAM Study population showed differences in the occurrence and some characteristics of these clusters. A recent systematic review summarizes data of 14 studies that have employed unsupervised learning methods to derive subgroups of adult-onset diabetes in populations from Asia, Europe, the USA, and Australia^10^. In five of them, the same five subgroups were extracted. While the obesity-related subgroup is also highly prevalent in previous studies (20–34%), this cluster amounted to 73% among the present Ghanaian sample. Further, the age-related subgroup constitutes the most common cluster in non-Ghanaian populations (34–45%). Yet, only 10% of individuals with aDM in the RODAM Study exhibited an age-related phenotype. Another difference is discernible for the insulin-resistant subgroup. This yielded 7–24% in previous studies but only 5% in the present population. These findings might be explained by the poor discriminative ability of BMI for diabetes mellitus among Ghanaians^26,27^ and the suggested large proportion of metabolically healthy obesity among African populations^29^. In fact, all subgroups in this study, except for the age-related cluster in the first approach, showed high BMI values and low HOMA-IR (Table 1). Further, the proportions of the insulin-deficient (7%) and the autoimmune subgroups (5%) in our study ranked at the lower end of the previously reported occurrences (3–22%). The subgroup of autoimmune diabetes mellitus might include individuals with LADA or type 1 diabetes mellitus. Yet, the concentrations of GAD65Ab and the proportions of autoantibody-positive individuals are similar between participants with and without aDM^22^. Hence, this biomarker may not discriminate well for diabetes status in this population, and the meaning of GADA positivity in the latter subgroup remains unclear. Further, the small proportion of the insulin-deficient subgroup may result from the presence of ketosis-prone diabetes, possibly hidden within the age-related cluster (Figure S1). A considerable proportion of individuals with aDM from sub-Saharan Africa have ketosis-prone diabetes, characterized by urinary ketone bodies and reserved beta-cell function after stabilization of blood glucose through initial insulin treatment^30^.

In fact, the second approach appears to separate these so-called atypical forms of diabetes mellitus from the conventional types. Many features of the ketosis-prone subgroup (6%) from the second approach accord with previously published features of this atypical phenotype: Age at onset was in the fourth decade, family history of diabetes was common, BMI, and body fat percentage were high, 6% of these patients presented with urinary ketones, half of the participants received insulin immediately at diagnosis, and beta-cell reserve was moderate (Table 1). In addition, the malnutrition-related sub-group (8%) exhibited characteristics of the previously described protein-deficient pancreatic diabetes and fibro-calculous pancreatic diabetes^30^: Age at onset was rather young, family history of diabetes was less prominent than in other clusters, BMI and body fat percentage were low, and beta-cell function was impaired. Notably, the malnutrition-related subgroup was more common in Ghana, and corroborates our hypothesis that environmental triggers may outweigh genetic predisposition in aDM^22^. Further, the obesity- and age-related subgroup presented as the largest cluster (68%). This subgroup resembled the obesity-related subgroup identified in the first approach, particularly mean BMI and mean HbA1c were high, while insulin resistance was moderate. The cluster characteristics largely overlapped with the body fat-related and insulin-resistant subgroup (18%), except for the high values of mean HOMA-IR in the latter. Therefore, the body fat-related and insulin-resistant subgroup is similar in proportion and features to SIRD derived in other populations^10^.

### Therapeutic strategies for glycaemic control and prevention of diabetic complications

Recently, Tanabe et al.^30^ have suggested therapeutic strategies for the observed subgroups along a continuum of insulin demand and secretory capacity for the prevention of diabetic complications. For insulin-deficient subgroups, including the autoimmune-related and the insulin-deficient clusters, insulin therapy appears to be the treatment of choice, possibly in combination with oral insulin secretagogues, to stabilize long-term blood glucose (HbA1c) and to prevent the development of retinopathy—the most common diabetic complication in these subgroups^12^. This is corroborated by our findings in the RODAM Study for both analysis approaches. Yet, we also noticed some discrepancies between our results and the previously reported treatment and complication profiles of those clusters characterized by insulin deficiency. Among Ghanaians, kidney dysfunction and PAD were also common in those subgroups characterized by high proportions of GAD65Ab; they were mainly prescribed oral glucose-lowering medication. This difference may stem from our cross-sectional study design and the inclusion of prevalent aDM cases with existing diabetic complications. Indeed, disease duration affects the cluster allocation, as individuals potentially transition from one subgroup to another over time^13^. This remains to be investigated when follow-up data of the RODAM study population will be available. For subgroups characterized by older age of disease onset, metformin and dipeptidyl peptidase-4 inhibitors (DPP4i) have been proposed to prevent the observed risks of coronary events^31^. In the RODAM Study population, similar treatment profiles and diabetic complications (stroke, CAD, PAD) were discernible in the age-related subgroup (first approach) and in the obesity- and age-related subgroup (second approach). Yet, lifestyle modifications were also frequently prescribed for the obesity- and age-related subgroup derived in the second approach, possibly due to the large proportion of individuals with insulin resistance and obesity. In other studies, this subgroup emerged as the obesity-related cluster with high BMI and frequent occurrence of liver dysfunction. For diseases management, literature suggests to emphasize on weight control with diet and physical exercise plus metformin, and other oral glucose-lowering medication^31^. This strategy is promoted even stronger for insulin-resistant subgroups, and comprise a combination of weight-loss through lifestyle and surgery, as well as insulin-sensitizing drugs for the prevention of microvascular complications, mainly kidney dysfunction^31^. Our results from the first approach confirm these treatment and complication profiles, whereas the body fat-related and insulin-resistant subgroup from the second approach was dominated by liver dysfunction and cardiovascular events.

### Strengths and limitations

This analysis uniquely adds to understanding the aetiology of aDM, and thereby, contributes to improved clinician counseling and pharmacological interventions. We provide evidence that subgroups of diabetes mellitus may be reproduced in sub-Saharan African populations, when using the previously published clinical variables and methodological approach. Clearly, our results need to be interpreted with caution, as we studied prevalent aDM with different disease durations. These factors might have influenced cluster allocation, proportions, and complication profiles. As a research prospect, we aim at verifying our results in the follow-up data, 5 years after baseline. Here, we have applied hierarchical clustering followed by k-means clustering, which are more suitable when aiming to separate subgroups. Other colleagues have used soft-clustering as an alternative^32^, which allows participants to represent in more than one subgroup—an approach that might have less clinical relevance but could facilitate the study of disease aetiology. Also, future work might expand to classify various states of dysglycemia, including impaired glucose tolerance and pre-diabetes. Reassuringly, the clusters in our study remained stable when individuals on insulin treatment were excluded from the analysis (Ghana-specific approach). Additional variables can aid the derivation of aDM subgroups, including metabolomics and genomics^9,10^. Yet, we refrained from these approaches as they are unlikely to be incorporated into clinical practice. Also, we acknowledge that HOMA-beta and HOMA-IR were based on fasting insulin, not on fasting C-peptide, which contrasts the methodology by Ahlqvist and colleagues. For HOMA-IR, insulin-based values may overestimate insulin resistance among individuals on insulin medication. Still, sensitivity analysis excluding participants on insulin treatment (16%) produced similar subgroups. For HOMA-beta, insulin-based calculations might underestimate beta-cell function due to hepatic insulin storages, but they reflect impaired hepatic insulin clearance, which likely contributes to insulin resistance^28^. In this cross-sectional study, we cannot comment on the stability of these clusters, as individuals can migrate over time and clinical features overlap between the identified subgroups. The RODAM Study population has been thoroughly phenotyped regarding biomarkers of glucose metabolism, anthropometric measurements, and several diabetic complications. Yet, we acknowledge that retinopathy, stroke and CAD were assessed by self-report only. Just as a large proportion of individuals with aDM from sub-Saharan Africa remains undiagnosed^3^, so do their diabetic complications. Therefore, self-reported complications might have underestimated the true proportions in our study population, particularly for microvascular complications, and thus, contributed to type II error. Similarly, the various oral glucose-lowering medications were not documented in this study; this limits the interpretation of treatment-related characteristics across subgroups.

## Conclusion

In this Ghanaian study population, the first approach (replication from Ahlqvist et al.^9^) provided subgroups that separated well between diabetic complications, but differentiated less sharp between phenotypes. This was the opposite for the second approach (Ghana-specific variables): It produced well-known diabetic phenotypes among African populations, which however, did not separate well for diabetic complications. Pending verification in prospective studies with incident aDM among sub-Saharan African populations, our findings suggest that cluster analysis based on routinely collected clinical data can contribute to the understanding of diabetes aetiology and possibly disease progression.

## Supplementary Information

Supplementary Information.

## Data Availability

The study protocol and statistical analysis plans were previously published^19^. Individual participant data will be shared with researchers in a deidentified or anonymized format upon submitting a research proposal and requesting data access to Prof. Charles Agyemang, UMC Amsterdam (c.o.agyemang@amsterdamumc.nl). Data will be made available for analyses as approved by the data access committee.
