# Supplementary material for: Subgroups of adult-onset diabetes: a data-driven cluster analysis in a Ghanaian population
Source: Sci Rep. 2023 Jul 4;13:10756. doi: 10.1038/s41598-023-37494-2 (PMC10319880; doi:10.1038/s41598-023-37494-2)
Supplement: Supplementary file 1 — Supplementary Information. [file 41598_2023_37494_MOESM1_ESM.docx]

**Supplementary Table S1: General characteristics of the RODAM study population (N = 5,898)**

| **Characteristics** | **Total**  **(n = 5,898)** | **Diabetes**  **(n = 541)** | **No diabetes**  **(n = 5357)** |
| --- | --- | --- | --- |
| **Socio-demographic characteristics** |  |  |  |
| Age (years) | 46.1 ± 11.9 | 53.2 ± 9.5 | 45.4 ± 11.9 |
| Sex (female, %) | 62.2 (3606) | 55.8 (239) | 62.9 (3367) |
| Study location (%) |  |  |  |
| Rural Ghana | 18.8 (1111) | 10.4 (56) | 19.7 (1055) |
| Urban Ghana | 24.7 (1455) | 25.0 (135) | 24.6 (1320) |
| Amsterdam | 27.7 (1633) | 32.0 (173) | 27.3 (1460) |
| London | 19.0 (1121) | 19.4 (105) | 19.0 (1016) |
| Berlin | 9.8 (578) | 13.3 (72) | 9.5 (506) |
| Length of stay in Europe (years) | 17.3 ± 8.8 | 21.8 ± 9.5 | 16.7 ± 8.6 |
| Migration generation (1st, %) | 98.4 (5798) | 99.6 (539) | 98.2 (5259) |
| Education (%) |  |  |  |
| Never/elementary | 34.2 (1930) | 33.1 (179) | 34.6 (1851) |
| Lower | 36.5 (2140) | 37.2 (201) | 36.2 (1939) |
| Intermediate | 18.3 (1080) | 18.7 (101) | 18.3 (979) |
| Higher | 11.0 (648) | 11.1 (60) | 11.0 (588) |
| Occupation (%) |  |  |  |
| Non-manual | 31.9 (1868) | 34.9 (189) | 31.3 (1679) |
| Manual | 68.1 (3981) | 64.9 (351) | 67.8 (3630) |
| Smoking (ever, %) | 10.3 (597) | 14.4 (78) | 9.7 (519) |
| **Clinical characteristics** |  |  |  |
| Adult-onset diabetes mellitus* (yes, %) | 9.2 (5898) | 100 (541) | 0 (5357) |
| Diabetes in family (yes, %) | 20.1 (1186) | 41.4 (224) | 18.0 (962) |
| Duration of diabetes (years) | 5.0 (1.0-6.0) | 5.0 (1.0-6.0) | n.a. |
| Fasting glucose (mmol/L) | 5.4 ± 1.8 | 8.6 ± 4.4 | 5.0 ± 0.6 |
| HbA1c (mmol/mol) | 38.3 ± 12.1 | 60.8 ± 24.6 | 36.0 ± 6.6 |
| HbA1c (%) | 5.7 ± 1.1 | 7.7 ± 2.2 | 5.4 ± 0.6 |
| GAD65Ab (U/mL) | 16.8 (5.5-35.4) | 13.4 (3.5-31.3) | 17.2 (5.7-35.8) |
| GAD65Ab positivity (yes, %) | 6.1 (357) | 5.4 (29) | 6.1 (328) |
| C-reactive protein (mg/L) | 0.7 (0.2-2.5) | 1.2 (0.3-4.3) | 0.7 (0.2-2.4) |
| Current fever (yes, %) | 4.9 (287) | 3.7 (20) | 5.0 (267) |
| History of fever, past 2 weeks (yes, %) | 15.3 (901) | 9.6 (52) | 15.9 (849) |
| ALAT (U/L) | 19.3 (14.9-25.7) | 20.9 (16.1-27.7) | 19.2 (14.9-25.5) |
| ASAT (U/L) | 31.4 (25.7-39.0) | 29.9 (24.2-36.9) | 31.6 (26.0-39.2) |
| ASAT/ALAT | 1.6 (1.3-2.0) | 1.4 (1.2-1.7) | 1.64 (1.33-2.01) |
| GGT (U/L) | 30.6 (23.0-42.8) | 37.9 (28.1-53.6) | 29.9 (22.7-41.6) |
| Serum creatinine (µmol/L) | 83.0 (71.7-95.7) | 84.8 (71.4-96.6) | 83.0 (71.8-95.7) |
| eGFR (ml/min/1.73 m^2^) | 0.53 (0.46-0.61) | 0.53 (0.45-0.59) | 0.53 (0.46-0.61) |
| Urinary albumin (mg/L) | 4.0 (4.0-11.8) | 4.0 (4.0-19.8) | 4.0 (4.0-11.1) |
| Microalbuminuria (yes) | 15.0 (888) | 24.6 (133) | 14.1 (755) |
| **Morphometric characteristics** |  |  |  |
| Body mass index (kg/m^2^) | 27.1 ± 5.5 | 28.7 ± 5.6 | 27.0 ± 5.4 |
| Overweight (yes, %) | 35.5 (2093) | 39.7 (215) | 35.1 (1878) |
| Obesity (yes, %) | 27.5 (1621) | 36.6 (198) | 26.6 (1423) |
| Waist circumference (cm) | 90.4 ± 12.7 | 97.1 ± 13.0 | 89.7 ± 12.5 |
| Abdominal obesity (yes, %) | 42.0 (2473) | 55.8 (302) | 40.5 (2171) |

Data are presented as mean ± standard deviations for normally distributed continuous variables, as median (interquartile ranges) for skewed continuous variables, and as percentage for categorical variables. *Defined as fasting plasma glucose ≥7 mmol/L or use of glucose-lowering medication or self-reported diabetes, and age of onset ≥18 years.

**Supplementary Table S2: Stability of diabetes subgroups derived by hierarchical cluster analysis, assessed by k-means analysis**

| **Type of cluster analysis** | **Clusters derived using six clinical and anthropometric variables according to Ahlqvist et al. 2018** | | | | |
| --- | --- | --- | --- | --- | --- |
|  | **Obesity-related** | **Age-related** | **Autoimmune-related** | **Insulin-deficient** | **Insulin-resistant** |
| **Hierarchical** |  |  |  |  |  |
| % (n) | 72.8 (394) | 10.4 (56) | 5.4 (29) | 6.5 (35) | 5.0 (27) |
| Distance between cluster centroids | 5.966 | 7.998 | 5.966 | 6.081 | - |
| **k-means** |  |  |  |  |  |
| % (n) | 69.5 (376) | 13.7 (75) | - | 12.6 (68) | 4.1 (22) |
| Distance between cluster centroids | 2.260 | 2.352 | - | 2.260 | 4.417 |
| **Type of cluster analysis** | **Clusters derived using four variables with clinical relevance for Ghanaian populations** | | | | |
|  | **Obesity- and  age-related** | **Malnutrition-related** | **Body fat-related and insulin-resistant** | **Ketosis-prone** |  |
| **Hierarchical** |  |  |  |  |  |
| % (n) | 67.6 (366) | 7.9 (43) | 18.1 (98) | 6.3 (34) |  |
| Distance between cluster centroids | 1.684 | 7.477 | 1.684 | 6.293 |  |
| **k-means** |  |  |  |  |  |
| % (n) | 62.5 (338) | 12.2 (66) | 17.9 (97) | 7.4 (40) |  |
| Distance between cluster centroids | 2.051 | 2.214 | 2.051 | 2.971 |  |

**Supplementary Table S3: Subgroups of adult-onset diabetes and their characteristics among 454 Ghanaian adults who were not on insulin treatment**

| **Characteristics** | **Cluster 1** | **Cluster 2** | **Cluster 3** | **Cluster 4** |
| --- | --- | --- | --- | --- |
|  | **Obesity- and age-related** | **Malnutrition-related** | **Body fat-related and**  **insulin-resistant** | **Ketosis-prone** |
| n | 183 | 178 | 80 | 13 |
| Age at diabetes diagnosis (years) | 50.4 ± 8.7 | 41.1 ± 15.4 | 49.8 ± 9.8 | 36.8 ±18.4 |
| Fasting glucose (mmol/L) | 7.0 (5.7-8.5) | 6.3 (5.1-7.6) | 14.2 (12.1-16.4) | 8.2 (7.7-9.7) |
| HbA1c (mmol/mol) | 52.6 (43.8-62.7) | 46.2 (39.9-54.7) | 84.1 (70.4-111.1) | 46.4 (40.8-53.6) |
| Insulin (mU/L) | 8.3 (5.5-12.3) | 5.6 (3.7-8.8) | 5.8 (3.8-8.7) | 42.0 (36.3-47.2) |
| HOMA-IR | 2.48 (1.38-4.01) | 2.51 (1.32-4.14) | 3.11 (1.92-6.62) | 3.90 (1.18-5.35) |
| HOMA-beta | 37.8 (17.0-75.7) | 34.6 (18.3-68.3) | 44.3 (23.6-61.5) | 19.8 (11.1-40.7) |
| Body Mass Index (kg/m2) | 32.0 ± 5.0 | 25.9 ± 4.2 | 25.8 ± 4.3 | 30.2 ± 7.9 |
| Waist circumference (cm) | 106.7 ± 9.6 | 88.9 ± 9.2 | 90.3 ± 10.6 | 99.8 ± 16.1 |
| GAD65Ab (positive) | 6.0 | 4.3 | 3.2 | 2.8 |

Data are shown as means ± standard deviations, medians (interquartile ranges) or percentages.

**Supplementary Table S4: Subgroups of adult-onset diabetes and their characteristics among 191 Ghanaian adults in Ghana and 350 Ghanaian adults in Europe**

| **Characteristics** | **Ghana (n = 191)** | | | |
| --- | --- | --- | --- | --- |
|  | **Obesity- and age-related** | **Malnutrition-related** | **Body fat-related and insulin-resistant** | **Ketosis-prone** |
| % (n) | 29.3 (56) | 14.1 (27) | 38.2 (73) | 18.3 (35) |
| Age at diabetes diagnosis (years) | 48.0 ± 12.2 | 46.9 ± 12.5 | 46.7 ± 12.8 | 48.7 ± 14.5 |
| Fasting glucose (mmol/L) | 8.6 (7.0-12.6) | 10.8 (7.6-12.7) | 8.9 (6.7-12.2) | 9.8 (7.0-13.8) |
| HbA1c (mmol/mol) | 65.8 (42.4-105.6) | 61.0 (38.9-97.4) | 62.9 (47.3-79.7) | 63.2 (46.4-85.2) |
| Insulin (mU/L) | 6.1 (3.9-8.4) | 5.6 (4.7-10.0) | 8.9 (4.8-13.1) | 7.2 (4.7-12.1) |
| HOMA-IR | 2.49 (1.23-4.75) | 3.07 (1.68-4.56) | 3.72 (1.97-6.32) | 3.32 (1.51-6.58) |
| HOMA-beta | 19.2 (10.7-47.2) | 19.2 (10.6-44.3) | 33.8 (15.4-57.7) | 30.0 (8.4-82.8) |
| Ketone bodies (positive) | 4.5 | 3.7 | 8.2 | 8.5 |
| Body Mass Index (kg/m2) | 25.2 ± 4.6 | 24.4 ± 5.9 | 27.8 ± 48 | 26.1 ± 7.1 |
| Waist circumference (cm) | 89.4 ± 11.4 | 87.0 ± 13.2 | 94.2 ± 11.8 | 90.3 ± 13.9 |
| GAD65Ab (positive) | 23.2 | 3.7 | 2.7 | 11.4 |
| **Characteristics** | **Europe (n = 350)** | | | |
|  | **Obesity- and age-related** | **Malnutrition-related** | **Body fat-related and insulin-resistant** | **Ketosis-prone** |
| % (n) | 47.1 (165) | 1.1 (4) | 32.0 (112) | 19.7 (69) |
| Age at diabetes diagnosis (years) | 45.4 ± 12.1 | 49.4 ± 10.2 | 44.5 ± 11.2 | 45.2 ± 14.5 |
| Fasting glucose (mmol/L) | 7.1 (5.6-8.6) | 16.2 (10.5-18.2) | 6.9 (5.6-8.8) | 7.0 (5.4-8.2) |
| HbA1c (mmol/mol) | 51.1 (43.8-61.0) | 121.4 (86.7-128.4) | 50.5 (43.4-59.9) | 52.9 (45.7-68.1) |
| Insulin (mU/L) | 7.1 (4.4-10.8) | 8.0 (6.3-8.4) | 6.5 (4.4-10.7) | 7.2 (4.4-11.9) |
| HOMA-IR | 2.17 (1.33-3.50) | 5.8 (3.3-6.8) | 2.02 (1.23-3.73) | 2.05 (1.18-3.95) |
| HOMA-beta | 41.1 (28.3-78.4) | 12.7 (11.4-27.2) | 47.2 (23.2-32.4) | 43.2 (24.1-69.1) |
| Ketone bodies (positive) | 2.4 | 0 | 1.0 | 1.5 |
| Body Mass Index (kg/m2) | 30.1 ± 4.7 | 30.3 ± 3.7 | 29.3 ± 5.4 | 30.5 ± 5.2 |
| Waist circumference (cm) | 100.9 ± 10.8 | 101.7 ± 11.2 | 98.7 ± 13.2 | 101.7 ± 12.3 |
| GAD65Ab (positive) | 1.2 | 0 | 2.7 | 5.8 |

Data are shown as means ± standard deviations, medians (interquartile ranges) or percentages.

**
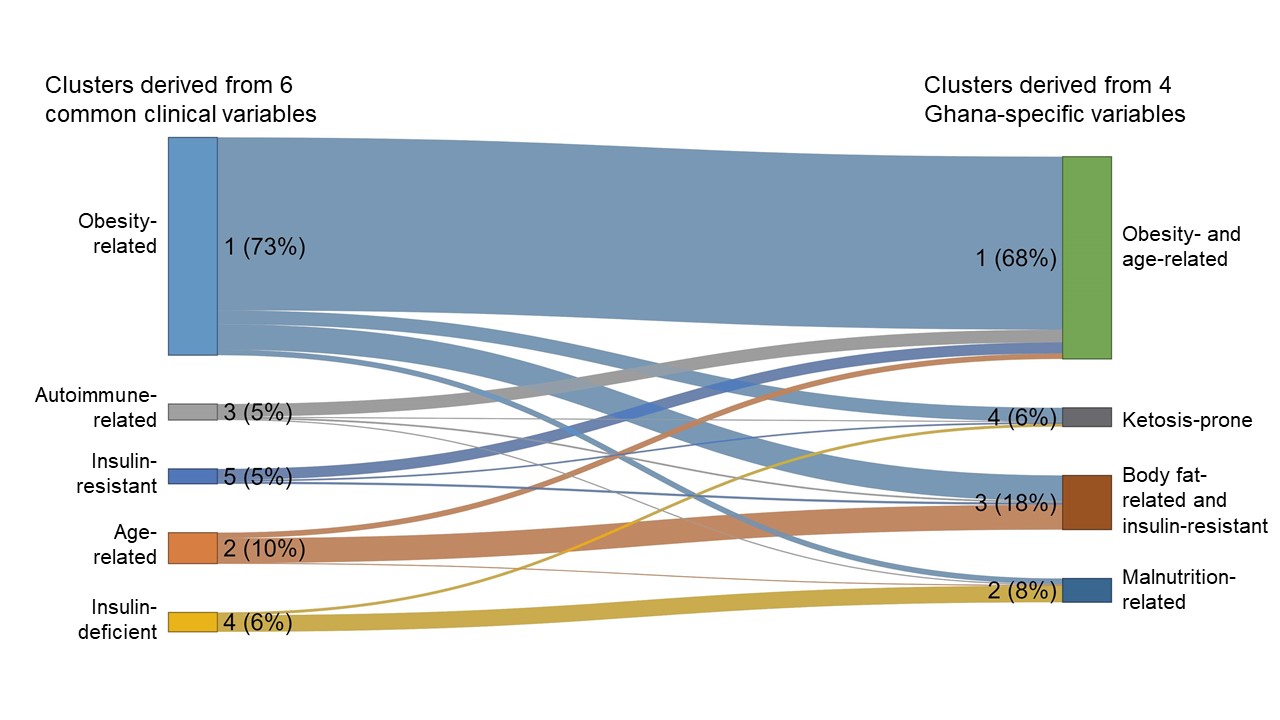
**

**Supplementary Figure S1: Overlaps of diabetes subgroups derived by two different approaches (left: cluster analysis using six common clinical and anthropometric variables; right: cluster analysis using four variables with clinical relevance for Ghanaian populations)**

| **A** | **B** |
| --- | --- |
|  |  |

**Supplementary Figure S2: Diabetes subgroups derived from cluster analysis using Ghana-specific biomarkers and stratified for place of living among (A) non-migrants in Ghana and (B) Ghanaian migrants in Europe**

| **A** |
| --- |
|  |
| **B** |
|  |

**Supplementary Figure S3: Complications in subgroups derived by cluster analysis using five variables with clinical relevance for Ghanaian populations among (A) non-migrants in Ghana and (B) Ghanaian migrants in Europe**

Note: There were only 4 individuals in the malnutrition-related subgroup in Europe.
